# Indirect Fist Percussion of the Liver Is a More Sensitive Technique for Detecting Hepatobiliary Infections than Murphy's Sign

**DOI:** 10.1155/2015/431638

**Published:** 2015-12-15

**Authors:** Takeshi Ueda, Eri Ishida

**Affiliations:** Emergency and General Internal Medicine, Rakuwakai Marutamachi Hospital, 9-7 Jyurakumawari-Matsushita-cho, Marutamachi, Nakagyo-ku, Kyoto 604-8401, Japan

## Abstract

*Background*. Murphy's sign and Charcot's triad are established clinical findings of acute cholecystitis and cholangitis, respectively, but both show low sensitivity and limited clinical application. We evaluated if indirect fist percussion of the liver improves the efficiency of diagnosing cholecystitis and cholangitis when used as a diagnostic adjunct.* Methods*. The presence/absence of right upper quadrant (RUQ) tenderness, Murphy's sign, and pain induced by indirect fist percussion of the liver was assessed, and the results were compared with the definite diagnosis based on ultrasound and additional examinations in patients aged over 18 who visited our outpatient clinic with suspected hepatobiliary diseases.* Results*. Four hundred and eight patients were investigated, and 40 had hepatobiliary infection (acute cholecystitis: 10, acute cholangitis: 28, liver abscess: 1, and hepatic cyst infection: 1). The sensitivity of indirect fist percussion of the liver for diagnosing hepatobiliary infection was 60%, being significantly higher than that of RUQ tenderness (33%) and Murphy's sign (30%), and its specificity was 85%. There was no significant improvement in sensitivity or diagnostic accuracy when Murphy's sign was combined with indirect fist percussion of the liver.* Conclusion*. Indirect fist percussion-induced liver pain is a useful clinical finding to diagnose hepatobiliary infection, with high-level sensitivity.

## 1. Introduction

Biliary tract infection causes abdominal pain and fever, and acute cholangitis is more likely to follow a severe clinical course and be associated with a higher mortality rate compared to acute cholecystitis [1]; therefore, it is important to diagnose it accurately.

Murphy's sign has been reported to be useful for diagnosing acute cholecystitis [2]. On the other hand, Charcot's triad, which is an established indicator of acute cholangitis, shows low-level diagnostic sensitivity [3], and diagnosing it mostly relies on laboratory and imaging examinations. In acute cholangitis, pain is known to be induced by striking the right hypochondrium with the hand in a fisted position (indirect fist percussion of the liver); however, its diagnostic accuracy has not been reported.

We evaluated the diagnostic usefulness of Murphy's sign and indirect fist percussion of the liver when examining patients with suspected hepatobiliary infection.

## 2. Methods

The design of the study was retrospective. The subjects were consecutive patients who visited the Outpatient Clinic of Emergency and General Internal Medicine of Rakuwakai Marutamachi Hospital from February 1, 2014, to March 31, 2015, and presented with fever, upper abdominal pain, a poor general condition, or hepatic dysfunction and were suspected to have hepatobiliary diseases. Abdominal ultrasound was performed after physical examination, including assessment of the presence of Murphy's sign, right upper quadrant (RUQ) tenderness, and indirect fist percussion of the liver. A definite diagnosis was made after laboratory examination, abdominal CT, magnetic resonance cholangiopancreatography (MRCP), and endoscopic retrograde cholangiopancreatography (ERCP) were performed if necessary, and the diagnostic utility of the physical findings was evaluated.

Hepatobiliary infections were defined as acute cholecystitis and cholangitis, liver abscess, and hepatic cyst infection. Diagnoses of acute cholecystitis and cholangitis were made based on the Tokyo Guidelines of 2013 (TG13) [4, 5]. Hepatobiliary diseases except for hepatobiliary infection were defined as diseases in which laboratory examination showed acute hepatic dysfunction. A doctor other than the attending physician reviewed the medical chart, and when the diagnosis was questionable, a definite diagnosis was determined after discussion with the attending physician.

Patients who were not able to complain of pain were excluded; however, when the patients showed escape movements or winced, it was judged that they perceived pain. Jaundice was considered present when the peripheral bulbar conjunctiva was yellowish. To assess Murphy's sign, the patient was placed in a supine position, and the tips of the examiner's left fingers were directed to the midline of the patient and placed at the base of the right front chest with the thumb on the lowest rib. The patient was instructed to inspire deeply while the examiner's thumb pressed into the patient's abdomen with the palmar side abducted. The test was considered positive if the patient's inspiration was interrupted due to pain. When the patient could not inspire deeply or inspiration was interrupted bilaterally, the test was considered indeterminate. Indirect fist percussion of the liver was performed with the examiner's left palm on the patient's right lower ribs, and the blow was delivered with the lateral aspect of the right hand. Pain was considered present when there was a difference between the right and left sides. RUQ tenderness was considered positive when the degree of tenderness was the highest in the right upper abdomen.

All statistical analyses were performed with EZR (Saitama Medical Center, Jichi Medical University, Saitama, Japan), which is a graphical user interface for R (The R Foundation for Statistical Computing, Vienna, Austria). More precisely, it is a modified version of R Commander designed to add statistical functions frequently used in biostatistics [6]. Ethical approval was granted by the Rakuwakai Marutamachi Hospital Ethics Committee.

## 3. Results

A total of 408 patients (194 males, 214 females) with a mean age of 70 (18–103) years were investigated. Fever, emesis, epigastralgia, RUQ pain, and jaundice were observed in 63.2, 12.5, 16.7, 6.6, and 1.0% of the patients, respectively. The definite diagnosis was hepatobiliary infection, other hepatobiliary diseases, and nonhepatobiliary diseases in 40, 65, and 303 patients, respectively (Table 1). Fifty-two percent of the nonhepatobiliary diseases were infectious diseases, such as pneumonia and urinary tract infection.

**Figure 1:**
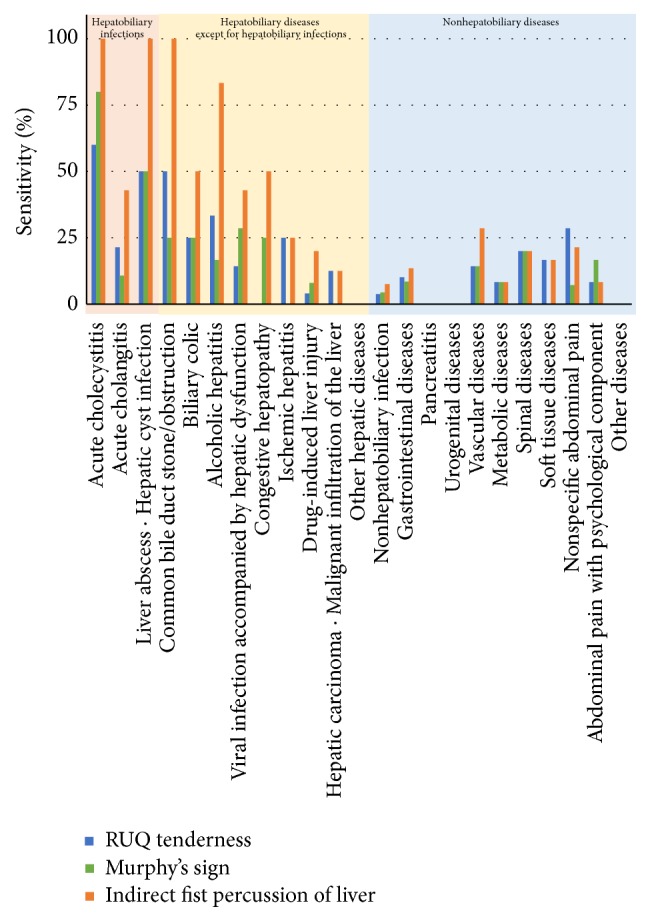
Diagnostic sensitivity. The sensitivity of indirect fist percussion of the liver was higher than that of Murphy's sign for acute cholecystitis and acute cholangitis. Indirect fist percussion of the liver tended to be positive in the presence of cholelithiasis, alcoholic hepatitis, viral hepatitis, and drug-induced hepatitis.

The sensitivity of Murphy's sign was 80% for cholecystitis, while that for cholangitis was 11%. The sensitivity of indirect fist percussion of the liver was 100 and 43% for cholecystitis and cholangitis, respectively, being higher than that of Murphy's sign (*p* = 0.042, Bonferroni correction) (Figure 1).

Indirect fist percussion of the liver tended to exhibit higher sensitivity compared to RUQ tenderness (*p* = 0.073, Bonferroni correction) and a significantly higher sensitivity than Murphy's sign for hepatobiliary infection (*p* = 0.039) (Table 2). In hepatobiliary diseases, a significantly higher sensitivity was noted compared to RUQ tenderness and Murphy's sign (*p* = 0.0007 and 0.0002, resp.) (Table 3).

Murphy's sign was considered indeterminate in 192 patients with insufficient deep inspiration due to disturbance of consciousness or the presence of dementia or mental disorders or with the interruption of deep inspiration as a result of tenderness involving the left hypochondrium. They were judged as negative for Murphy's sign, but in an analysis excluding these cases, of 216 patients in whom all physical findings were obtained (complete dataset), indirect fist percussion of the liver had a higher sensitivity compared to RUQ tenderness and Murphy's sign, and the differences were significant in diagnosing hepatobiliary diseases (*p* = 0.002 and 0.019, resp.).

**Figure 2:**
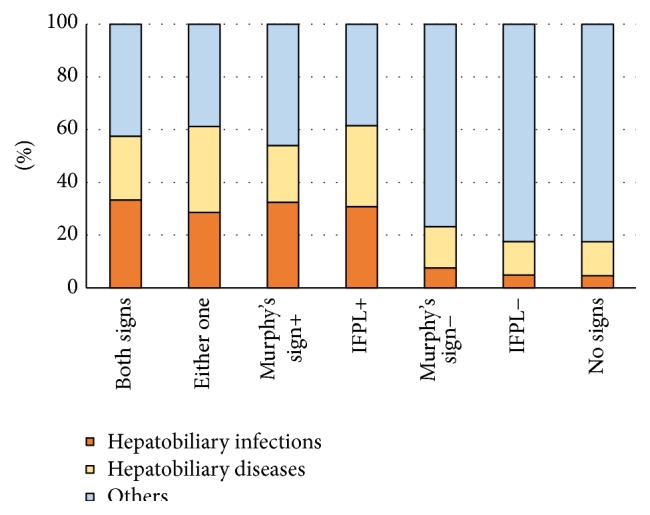
Likelihood of hepatobiliary diseases estimated by Murphy's sign and indirect fist percussion of the liver. The likelihood of hepatobiliary diseases estimated by indirect fist percussion of the liver alone was almost the same as that estimated based on indirect fist percussion of the liver in combination with Murphy's sign. IFPL: indirect fist percussion of the liver.

In multivariate analysis, indirect fist percussion of the liver, Murphy's sign, and RUQ tenderness showed a decreasing correlation with hepatobiliary infection and disorders in that order. The likelihood of hepatobiliary diseases estimated by indirect fist percussion of the liver and Murphy's sign is shown in Figure 2. If either one was positive, the likelihood of hepatobiliary infection was 28.6%, and that of hepatobiliary infection and disorders was 61.2%. On the other hand, if both of them were negative, the likelihood of hepatobiliary infection was 4.6%, and that of hepatobiliary infection and diseases was 17.5%. These values were similar to those when indirect fist percussion of the liver was negative (4.8 and 17.6%, resp.) and lower than those when Murphy's sign was negative (7.5 and 23.2%, resp.).

## 4. Discussion

This study suggested that indirect fist percussion of the liver is useful for diagnosing hepatobiliary infection and diseases, with a higher sensitivity than Murphy's sign. The maneuver is straightforward and can be performed for patients with dementia and mild disturbance of consciousness, unlike assessment for Murphy's sign.

A meta-analysis showed that the sensitivity and specificity of Murphy's sign for cholecystitis were 65% (58–71) and 87% (85–89) [7], and those of RUQ tenderness were 77% (73–81) and 54% (52–56), respectively. Acute cholecystitis was present in only 10 patients in this study, but indirect fist percussion-induced liver pain was observed in all of them, while Murphy's sign and RUQ tenderness were present in 80 and 60%, respectively, suggesting that indirect fist percussion of the liver shows a high diagnostic sensitivity for acute cholecystitis. It has been reported that the sensitivity of Murphy's sign was low (48%) in elderly patients (mean age: 79 years) with acute cholecystitis [8]. However, indirect fist percussion of the liver was positive in all 8 patients aged over 70 with acute cholecystitis in this study: its sensitivity for acute cholecystitis in elderly patients may also be high.

The rate of severe cases is higher in those with acute cholangitis (11.6%) [3] compared with acute cholecystitis (6.0%) [9], and the mortality rate due to acute cholangitis is 2.7% [10], being higher than that associated with cholecystitis (0.6%) [11]. Therefore, it is particularly important to diagnose acute cholangitis among all hepatobiliary infections.

Concerning cholangitis, Charcot described a triad of findings in 1877 [12] (Charcot's triad): (1) jaundice, (2) fever, usually with rigors, and (3) RUQ pain.

A study of 412 patients with acute cholangitis from 1963 to 1983 reported that Charcot's triad was observed in 72% [13]. Many cases of acute cholangitis inconsistent with Charcot's triad were subsequently reported, and a study in 1992 stated that Charcot's triad was present in only 22% of 512 acute suppurative cholangitis patients [14]. A recent study showed that although Charcot's triad shows very high-level specificity (95%) and its presence strongly suggests acute cholangitis, the sensitivity is only 26.4%, and it is considered that Charcot's triad is not applicable for ruling out a diagnosis [3]. In this study, none of the 40 patients with hepatobiliary infection met the criteria of Charcot's triad.

The Tokyo Guidelines for the management of acute cholangitis and cholecystitis (TG13) state that if one item from each category below is met, a diagnosis of acute cholangitis can be made with a sensitivity of 91.8% and specificity of 77.7%: (A) systemic inflammation (fever and/or shaking chills or laboratory evidence of an inflammatory response), (B) cholestasis (jaundice or laboratory data: abnormal liver function tests), and (C) imaging (biliary dilatation or etiological evidence on imaging (stricture, stone, stent, etc.) [3]. These guidelines are excellent with high-level sensitivity; however, they do not incorporate physical findings on abdominal examination and largely rely on laboratory and imaging examinations. Therefore, if we rely on them, such examinations need to be performed for all patients with fever to rule out acute cholangitis.

Physical examination of the abdomen is considered necessary to judge the necessity of adding laboratory and imaging examinations. In the present study, indirect fist percussion of the liver had a higher sensitivity for not only acute cholangitis but also cholecystitis compared to Murphy's sign, and it is considered to be useful for determining which patients require additional examinations. Furthermore, indirect fist percussion of the liver may have a high sensitivity for biliary colic (Figure 1).

In this study, Murphy's sign was considered indeterminate in 197 patients (47.1%). Four reasons can be speculated: (1) the study population included many elderly people with dementia, who could not follow instructions sufficiently; (2) it included many with disturbed consciousness due to severe infection (urinary infection, etc.); (3) it included 52 with pneumonia and 7 with decompensated heart failure, respectively, and so thorough evaluation of deep inspiration may have been difficult; (4) nonspecific abdominal pain or that with a psychological component can present as bilateral Murphy's sign as the site of abdominal pain is not fixed. Although laterality is generally not assessed, Murphy's sign present bilaterally was considered indeterminate to distinguish between a true positive sign and “nonspecific” positive sign due to functional abdominal pain. The test was considered indeterminate in 13 out of 26 patients diagnosed with nonspecific abdominal pain or that with a psychological component. Indeterminate Murphy's sign was analyzed as negative, but, to establish its validity, we also conducted an analysis only including patients in whom the presence/absence of Murphy's sign was determined (complete dataset), confirming the absence of significant differences in diagnostic accuracy.

Indirect fist percussion of the liver is considered to be more useful for diagnosing hepatobiliary infection and diseases than Murphy's sign, and it can be performed in patients with dementia or mild consciousness disturbance. Furthermore, its usefulness to diagnose hepatobiliary diseases is similar when combined with Murphy's sign (Figure 2). Therefore, we propose its use as a screening examination for patients presenting with fever or abdominal pain.

A study limitation was that the number of patients was low, at 40. The further accumulation of cases, including a larger number of younger patients, is required to verify the usefulness of indirect fist percussion of the liver.

## 5. Conclusion

In this study, it was suggested that indirect fist percussion of the liver is useful for diagnosing hepatobiliary infection and diseases, with a higher sensitivity than Murphy's sign. The percussion maneuver is very straightforward, and, unlike assessment for Murphy's sign, it can be performed for patients with dementia and mild disturbance of consciousness.

## Figures and Tables

**Table 1:** Definite diagnoses.

	Number of cases
Hepatobiliary infection	**40**
Cholecystitis	10
Cholangitis	28
Liver abscess · Hepatic cyst infection	2
Other hepatobiliary diseases	**65**
Common bile duct stone/obstruction	4
Biliary colic	4
Alcoholic hepatitis	6
Viral infection accompanied by hepatic dysfunction	7
Congestive hepatopathy	4
Ischemic hepatitis	4
Drug-induced liver injury	25
Hepatic carcinoma · Malignant infiltration of the liver	8
Other hepatic diseases	3
Other diseases	**303**
Nonhepatobiliary infection	159
Pneumonia	52
Urinary tract infection	71
Gastrointestinal diseases	59
Pancreatitis	5
Urogenital diseases	8
Vascular diseases	7
Metabolic diseases	12
Spinal diseases	5
Soft tissue diseases	12
Nonspecific abdominal pain	14
Abdominal pain with psychological component	12
Other diseases	10

**Table 2:** Diagnostic accuracy for hepatobiliary infection.

	Sensitivity	Specificity	LR+	LR−
RUQ tenderness	33 (19–49)	91 (88–94)	3.6 (2.1–6.3)	0.74 (0.60–0.92)
Under 65 y.o.	71 (29–96)	87 (80–92)	5.5 (2.9–11)	0.33 (0.10–1.1)
65–79 y.o.	60 (15–95)	92 (84–97)	7.9 (2.8–23)	0.43 (0.15–1.3)
Over 80 y.o.	20 (8–39)	93 (89–96)	2.9 (1.2–6.9)	0.86 (0.72–1.0)
Complete dataset	27 (12–46)	87 (81–91)	2.0 (0.99–4.0)	0.85 (0.68–1.1)
Murphy's sign	30 (17–47)	93 (90–96)	4.4 (2.4–8.1)	0.75 (0.61–0.92)
Under 65 y.o.	43 (10–82)	94 (88–97)	6.6 (2.2–20)	0.61 (0.32–1.2)
65–79 y.o.	40 (5–85)	96 (89–99)	11 (2.2–49)	0.62 (0.30–1.3)
Over 80 y.o.	23 (10–42)	93 (89–96)	3.4 (1.5–7.7)	0.82 (0.67–1.0)
Complete dataset	40 (23–59)	87 (81–91)	3.0 (1.7–5.3)	0.69 (0.52–0.93)
Indirect fist percussion of liver	60 (43–75)	85 (81–89)	4.1 (2.9–5.8)	0.47 (0.32–0.69)
Under 65 y.o.	57 (18–90)	82 (74–88)	3.1 (1.5–6.5)	0.53 (0.22–1.2)
65–79 y.o.	60 (15–95)	90 (81–96)	5.9 (2.2–16)	0.45 (0.15–1.3)
Over 80 y.o.	57 (37–75)	87 (82–91)	4.4 (2.7–7.1)	0.50 (0.33–0.75)
Complete dataset	57 (37–75)	77 (71–83)	2.5 (1.6–3.8)	0.56 (0.37–0.85)

**Table 3:** Diagnostic accuracy for hepatobiliary diseases.

	Sensitivity	Specificity	LR+	LR−
RUQ tenderness	21 (14–30)	92 (88–95)	2.6 (1.5–4.5)	0.86 (0.78–0.95)
Under 65 y.o.	33 (17–53)	89 (81–94)	3.1 (1.4–6.5)	0.75 (0.58–0.97)
65–79 y.o.	23 (8–45)	94 (84–98)	3.5 (1.0–12)	0.83 (0.65–1.0)
Over 80 y.o.	13 (6–24)	93 (88–96)	1.8 (0.78–4.3)	0.94 (0.84–1.0)
Complete dataset	22 (13–33)	88 (82–93)	1.8 (0.99–3.4)	0.89 (0.77–1.0)
Murphy's sign	19 (12–28)	94 (91–97)	3.4 (1.8–6.2)	0.86 (0.78–0.95)
Under 65 y.o.	20 (8–39)	95 (89–98)	4.0 (1.3–12)	0.84 (0.70–1.0)
65–79 y.o.	18 (5–40)	98 (91–100)	11 (1.3–95)	0.83 (0.68–1.0)
Over 80 y.o.	16 (8–28)	94 (89–97)	2.5 (1.1–5.6)	0.90 (0.80–1.0)
Complete dataset	27 (18–39)	88 (82–93)	2.3 (1.3–4.1)	0.82 (0.71–0.96)
Indirect fist percussion of liver	45 (36–55)	90 (86–93)	4.6 (3.1–6.8)	0.61 (0.51–0.73)
Under 65 y.o.	57 (37–75)	90 (83–95)	5.7 (2.9–11)	0.48 (0.32–0.73)
65–79 y.o.	32 (14–55)	94 (84–98)	4.9 (1.6–15)	0.73 (0.54–0.98)
Over 80 y.o.	40 (28–54)	89 (84–94)	3.8 (2.2–6.5)	0.67 (0.54–0.82)
Complete dataset	51 (39–63)	85 (78–90)	3.3 (2.1–5.1)	0.58 (0.46–0.74)
